# Assessing the Efficacy of Mental Health Assessment Training for Accredited Social Health Activists Workers in Rural India: A Pilot Study

**DOI:** 10.7759/cureus.37855

**Published:** 2023-04-19

**Authors:** Pooja Kasturkar, Sr. Tessy Sebastian, Jaya Gawai, Kavita P Dukare, Trupti Uke, Mayur B Wanjari

**Affiliations:** 1 Department of Mental Health Nursing, Smt. Radhikabai Meghe Memorial College of Nursing, Datta Meghe Institute of Higher Education and Research, Wardha, IND; 2 Department of Obstetrics and Gynecological Nursing, Smt. Radhikabai Meghe Memorial College of Nursing, Datta Meghe Institute of Higher Education and Research, Wardha, IND; 3 Department of Research and Development, Jawaharlal Nehru Medical College, Datta Meghe Institute of Higher Education and Research, Wardha, IND

**Keywords:** maharashtra, mental health assessment training program, asha (accredited social health activists), global mental health assessment tool-primary care marathi android version (gmhat-pc marathi android version), mental health issues

## Abstract

Background

Mental health issues are a major concern in rural India, but the lack of trained professionals limits access to care. In this pilot study, we evaluated the efficacy of a mental health assessment training program for Accredited Social Health Activists (ASHA) in rural Maharashtra, India.

Aim and objective

To conduct a pilot study to evaluate the feasibility and potential efficacy of Mental Health Assessment Training by using the Global Mental Health Assessment Tool-Primary Care Marathi Android version (GMHAT/PC-M) on ASHA workers in the Wardha district to identify mental health problems.

Methods

The study enrolled 12 ASHA workers from two rural health centers in Maharashtra. The workers completed a pretest and then received training in mental health assessment using the GMHAT/PC Marathi Android version. Mental health knowledge and global mental health assessment tool checklist scale scores were assessed on day seven, month one, and month three after training.

Results

The ASHA workers had a mean age of 42.2 years and a mean experience of 9.6 years. The majority were Hindus (50%), with the remaining workers being Buddhist. Of the 12 workers, only four had prior mental health training. Mental health knowledge and global mental health assessment tool checklist scale score significantly improved from the pretest to day seven (p<0.001), and the scores continued to improve at month one and month three with a 0.001 level of significance. At the end of the study, the mean mental health knowledge score was 15.2 (out of 20), and the mean global mental health assessment tool checklist scale score was 55.5 (out of 60).

Conclusion

Our pilot study demonstrated the effectiveness of a mental health assessment training program for ASHA workers in rural Maharashtra, India, using the GMHAT/PC Marathi Android version. The training program improved the mental health knowledge and GMHAT checklist scale of the ASHA workers, suggesting that such programs can help bridge the gap in mental health care in rural areas. Further studies with larger sample sizes and longer follow-up periods are needed to confirm the effectiveness of this training program.

## Introduction

Mental health disorders are a significant public health concern worldwide, with approximately one in four people affected at some point [1]. In low- and middle-income countries (LMICs), mental health disorders profoundly impact individuals, families, and communities. However, the resources to address these issues are often scarce, particularly in rural areas [2]. The situation is particularly challenging in India, where the prevalence of mental health disorders is estimated at 10-20% of the population [3]. Still, there is a severe shortage of mental health professionals, particularly in rural areas [4].

In India, the Accredited Social Health Activist (ASHA) program was launched in 2005 to improve the health of rural populations by providing a link between community members and the health system [5]. ASHAs are female community health workers who are trained to provide basic health services, health education, and referrals to primary health care facilities. However, ASHAs are not trained to identify or manage mental health disorders, despite the high prevalence of these disorders in rural areas. As a result, there is a need to develop and implement mental health training programs for ASHAs to improve their capacity to identify and manage mental health disorders.

In recent years, several studies have evaluated mental health training programs for community health workers in LMICs [6-9]. However, these studies have primarily focused on training programs for non-specialist health workers, such as lay counselors or community health volunteers. Few studies have evaluated mental health training programs specifically for ASHAs in India. Moreover, most of the studies have used self-reported measures to assess the effectiveness of the training programs, which may be subject to social desirability bias and may not accurately reflect changes in knowledge or skills [10].

To address this gap, we conducted a pilot study to evaluate the effectiveness of a mental health assessment training program for ASHAs in rural India using the Global Mental Health Assessment Tool-Primary Care Marathi Android version (GMHAT/PC-M) [11]. The GMHAT/PC-M is a validated tool used to assess mental health disorders in primary care settings in LMICs [12]. We used a pre- and post-test design to evaluate changes in mental health knowledge and GMHAT/PC-M scores among ASHAs who received the training program.

This pilot study is a crucial first step in developing and implementing effective mental health training programs for ASHAs in rural India. By improving the capacity of ASHAs to identify and manage mental health disorders, we can improve the health outcomes of individuals and communities in rural areas and reduce the burden of mental health disorders in India.

## Materials and methods

Study setting and design

The single-arm interventional study was conducted at the Primary Health Center (PHC) in Seloo, situated in the Wardha district of Maharashtra, India, between April to June 2022.

Study population

A total of 12 ASHA workers were included in this pilot study. The study included ASHA workers willing to participate and could read, write, comprehend Marathi, and use an Android phone. However, individuals who had already participated in a similar study within the past six months were excluded, as were those who did not use an Android phone.

Training program

The training program for mental health assessment aimed at ASHA workers included several modules. These modules included an introduction to mental health, recognizing the signs of a mentally healthy person, understanding the causes and symptoms of mental illness, common mental disorders along with their associated symptoms, an overview of GMHAT, identification of common mental disorders through GMHAT, and a practical demonstration. Furthermore, the program covered the referral system for patients with mental illnesses and a review of common mental disorder identification using GMHAT by ASHA workers.

Data collection

A structured knowledge questionnaire on mental health assessment was utilized to evaluate participants' knowledge. The questionnaire was designed to cover all aspects of mental health assessment, including the signs and symptoms of various mental disorders, the appropriate methods for conducting a mental health assessment, and the referral process and validation from experts in the subject area (Appendix 1).

In addition to the knowledge questionnaire, a checklist based on the global mental health assessment tool checklist scale was utilized to evaluate participants' skills. The checklist included various practical scenarios related to mental health assessment, and participants were required to demonstrate their skills by performing the appropriate actions for each scenario (Appendix 2).

The mental health assessment training was divided into a three-day module, which covered the following topics:

On day one, participants were given a pre-test to assess their baseline knowledge of mental health assessment. They were also provided background information on mental disorders, per the Global Mental Health Assessment Tool - Primary Care Version (GMHAT/PC) Marathi guidelines. Additionally, participants were given instructions on how to use the tool and rate the symptoms, as well as an overview of the referral system.

On day two, participants observed a demonstration of mental health assessment through GMHAT/PC Marathi. This gave them a clear understanding of how to conduct a mental health assessment using the tool and an opportunity to ask questions and seek clarification.

On day three, participants had the opportunity to perform a redemonstration of their mental health assessment skills using the GMHAT/PC Marathi tool. This provided them with an opportunity to practice and improve their skills, as well as receive feedback from their peers and trainers. We were contacted t the ASHA workers monthly meeting to the PHC Seloo Wardha. 

The scoring system for the knowledge questionnaire tool has been classified into four levels, as follows: Poor (0-5), Average (6-10), Satisfactory (11-15), and Very Good (16-20). The scoring for the global mental health assessment tool checklist was as follows: Poor (0-15), Below Average (16-30), Average (31-45), and Very Good (46-60).

The knowledge questionnaire consists of a total of 20 questions. Each question is assigned a score of one for the correct option and zero for all incorrect options. The total score for all 20 questions is then categorized as follows: Poor (0-5), Average (6-10), Satisfactory (11-15), and Very Good (16-20).

The global mental health assessment tool checklist comprises 15 questions, each of which is assigned a score category of Never (0), Once in a while (1), Sometimes (2), Most of the time (3), or Always (4). The total score for all 15 questions is categorized as follows: Poor (0-15), Below Average (16-30), Average (31-45), and Very Good (46-60).

Statistical analysis

We analyzed demographic variables of ASHA workers such as age, education, marital status, and monthly income. Descriptive statistics were used to calculate ASHA workers' knowledge and skill scores before and after Mental Health Assessment training, with mean and standard deviation. Paired t-tests were used to compare pre and post-test scores. To evaluate the efficacy of the training, repeated measures Analysis of Variance (ANOVA) was used to compare scores at seven days, one month, and three months post-training. We used SPSS version 26 (IBM Corp., Armonk, NY, USA) and considered a p-value <0.05 significant. Results are presented in tables and graphs.

Ethical consideration

Written informed consent was obtained from each participant after a careful explanation of the concept and purpose of the study. The study was approved by the Institutional Ethics Committee of Datta Meghe Institute of Medical Sciences (Deemed to be University) granted ethical approval (IEC/DEC-2019/8543).

## Results

Demographic characteristics 

Table 1 presents the demographic characteristics of the 12 ASHA workers who participated in the study. The table includes information on their age, religion, educational status, type of family, marital status, total family members, population served, experience in years, income, a family member suffering from mental illness, and training regarding mental health and mental illness.

**Table 1:** Demographic characteristics of the participant Mean (SD) for continuous; n (%) for categorical

Variable	N = 12
Age in years, Mean (SD)	42.2 (6.8)
Religion, No. (%)	
Hindu	6 (50.0)
Muslim	0 (0)
Christians	0 (0)
Buddhist	6 (50.0)
Others	0 (0)
Educational Status, No. (%)	
8^th^ Standard	3 (25.0)
9^th^ Standard	3 (25.0)
10^th^ Standard	5 (41.7)
More than 10^th^ Standard	1 (8.3)
Type of Family, No. (%)	
Nuclear Family	12 (100.0)
Joint Family	0 (0)
Extended Family	0 (0)
Marital Status, No. (%)	
Married	9 (75.0)
Widow	2 (16.7)
Divorced	0 (0)
Separated	1 (8.3)
Total family member, Mean (SD)	4.0 (0.7)
Population served, No. (%)	
1000 population	10 (83.3)
More than 1000 population	2 (16.7)
Experience in years, Mean (SD)	9.6 (4.3)
Income, Mean (SD)	30,416.7 (5,333.6)
Family member suffering from Mental Illness, No. (%)	
Yes	0 (0)
No	12 (100.0)
Training regarding mental health and mental illness, No. (%)	
Yes	4 (33.3)
No	8 (66.7)

Table 2 shows the mental health knowledge scores of the ASHA workers at different time points - pretest, day seven, month one, and month three. The table shows the number of participants and the percentage of their scores falling under different categories - poor, average, satisfactory, and very good.

**Table 2:** Mental health knowledge score at different time points

Mental Health Knowledge Score	Time Points				Total	Chi2	p-value
	Pretest (n=12)	Day 7 (n=12)	Month 1 (n=12)	Month 3 (n=12)			
Poor	2 (16.67%)	0 (0%)	0 (0%)	0 (0%)	2 (4.17%)	50.247	0.001
Average	10 (83.33%)	0 (0%)	0 (0%)	0 (0%)	10 (20.83%)		
Satisfactory	0 (0%)	6 (50%)	9 (75%)	8 (66.67%)	23 (47.92%)		
Very Good	0 (0%)	6 (50%)	3 (25%)	4 (33.33%)	13 (27.08%)		
Total	12 (100%)	12 (100%)	12 (100%)	12 (100%)	48 (100%)		

Table 3 shows the global mental health assessment tool checklist scale at different times, including the pretest, day seven, month one, and month three. The total number of participants was 12 for each time point. The table shows the distribution of participants according to their global mental health score, which was categorized as poor, average, satisfactory, and very good.

**Table 3:** Global mental health assessment tool checklist scale at different time points.

Global Mental Health Assessment Tool Checklist Scale	Time Points				Total	p-value
	Pretest (n=12)	Day 7 (n=12)	Month 1 (n=12)	Month 3 (n=12)		
Poor	10 (83.33%)	0 (0%)	0 (0%)	0 (0%)	10 (20.83%)	0.001
Average	2 (16.67%)	2 (16.67%)	1 (8.33%)	1 (8.33%)	6 (12.50%)	
Satisfactory	0 (0%)	0 (0%)	0 (0%)	0 (0%)	0 (0%)	
Very Good	0 (0%)	10 (83.33%)	11 (91.67%)	11 (91.67%)	32 (66.67%)	
Total	12 (100%)	12 (100%)	12 (100%)	12 (100%)	48 (100%)	

Table 4 shows the mean scores of mental health knowledge and the GMHAT at different time points. The study was conducted with 48 ASHA workers, and their scores were recorded at four different time points, including the pretest, day seven, month one, and month three. The mental knowledge and global mental health assessment tool checklist scale scores were recorded for each time point, and the mean and standard deviation (SD) were calculated.

**Table 4:** Mean scores of mental health knowledge and Global Mental Health Assessment Tool Checklist Scale at different time points

Variable	Time Points				
	Overall (n=48)	Pretest (n=12)	Day7 (n=12)	Month1 (n=12)	Month 3 (n=12)
Mental Health knowledge score, Mean (SD)	13.2 (3.7)	7.3 (1.5)	15.7 (1.2)	14.8 (1.0)	15.2 (1.6)
Global Mental Health Assessment Tool Checklist Scale, Mean (SD)	43.3 (22.6)	7.2 (14.8)	54.3 (6.1)	56.2 (4.0)	55.5 (4.0)

## Discussion

This pilot study evaluated the effectiveness of a mental health assessment training program for ASHAs in rural India using the GMHAT/PC-M. The results showed a significant improvement in mental health knowledge and GMHAT/PC-M scores among ASHAs who received the training program. Our study is one of the few to evaluate a mental health training program specifically for ASHAs in India. It provides important insights into the effectiveness of such programs in improving the capacity of ASHAs to identify and manage mental health disorders in rural areas.

Our study found that the mental health assessment training program effectively improved the knowledge and skills of ASHAs in identifying and managing mental health disorders. The GMHAT checklist scores of ASHAs who received the training program showed a significant improvement in identifying and managing mental health disorders. These findings are consistent with previous studies that have evaluated mental health training programs for non-specialist health workers in LMICs [6-9]. The results of our study suggest that mental health training programs for ASHAs in rural India can be an effective strategy for improving the capacity of primary healthcare workers to identify and manage mental health disorders.

The GMHAT/PC-M is a validated tool used to assess mental health disorders in primary care settings in LMICs [12]. Our study used the GMHAT checklist to assess the effectiveness of the mental health assessment training program in rural India. Using a validated tool to assess changes in mental health knowledge and skills provides important evidence for the effectiveness of the training program.

The ASHA program was launched in 2005 to improve the health of rural populations in India by providing a link between community members and the health system [5]. However, ASHAs are not trained to identify or manage mental health disorders, despite the high prevalence of these disorders in rural areas. Our study suggests that mental health training programs for ASHAs can be an effective strategy for improving the capacity of primary healthcare workers to identify and manage mental health disorders.

Our findings are consistent with previous studies that have evaluated the effectiveness of mental health training programs for non-specialist health workers in LMICs. For example, a study conducted in Kenya found that a brief training program for lay counselors improved their ability to identify and manage depression [13]. Another study in Pakistan found that a community-based mental health intervention led by trained community health workers improved participants' mental health outcomes [14]. These studies suggest that community health workers can play an important role in addressing the burden of mental health disorders in LMICs.

Finally, our study highlights the potential for mobile health (mHealth) technologies to support mental health training programs for ASHAs in rural India. The GMHAT/PC-M tool used in our study is an Android app, allowing for easy and efficient assessment of mental health disorders in primary care settings. Using mHealth technologies could also support ongoing supervision and support for ASHAs, such as messaging platforms or video conferencing. Several studies have demonstrated the potential for mHealth technologies to support mental health service delivery in LMICs [15]. Our study suggests these technologies could also support mental health training programs for ASHAs in rural India.

Limitations

Our study has several limitations that should be considered. First, the study was conducted in a single district in Maharashtra, India, which may limit the generalizability of the findings to other regions of India or LMICs. Second, the study used a pre-and post-test design, which may be subject to testing effects and social desirability bias. Third, the study did not evaluate the long-term effectiveness of the training program or its impact on mental health outcomes. Future studies should address these limitations by conducting multi-site studies with longer follow-up periods and incorporating measures of mental health outcomes.

## Conclusions

The study found that the training program was effective in improving the knowledge and skills of ASHAs in identifying and managing mental health disorders. Improving the capacity of ASHAs to identify and manage mental health disorders can improve the health outcomes of individuals and communities in rural areas and reduce the burden of mental health disorders in India. Further research is needed to evaluate the long-term impact of mental health training programs for ASHAs and to develop sustainable models for scaling up such programs in rural areas.

## Appendices

Appendix 1

Knowledge Questionnaire

1. Mentally ill person can be identified by a change in

a. Behaviour

b. Looks

c. Shouting                 

d. Laughing

2. A person is excessively happy without any reason. This is a sign of

a. Normal behviour                         

b. Being happy for others                   

c. Being happy for self                     

d. Abnormal behavior

3. Blaming self again and again for trivial things is a sign of  

a.  Aggression                                   

b. Depression              

c.  Personality problems                   

d. Anxiety

4. A person has lost job. This person needs to be watched for signs of

a.  Mania                                           

b. Financial burden                 

c. Depression                       

d. Personality problems

5. A physically healthy person will

a. Not have Mental illness               

b. May have mental illness    

c. Never drink alcohol                      

d. Never become angry

6. A mentally ill person will

a. Not have physical illness             

b. May get physical illness    

c. Be locked in a room                     

d. Never become angry

7. Making adjustments to lead a peaceful life is a sign of

a. Mental Illness                              

b. Mental Health        

c. Physical Health                            

d. Physical illness

8. The thinking process is altered in  

a. Mental Illness                              

b. Mental Health        

c. Physical Health                            

d. Physical illness

9. The mentally ill person may report of seeing things 

a. That are visible                            

b. Invisible     

c. That do not exist                           

d. Possessed by others

10. Excessive worrying interferes with

a. Day to day thinking                     

b.Dday to day activities          

c. Day-to-day observation                 

d. Daily interaction with others

11. Excessive sweating and palpitations are signs of   

a. Anxiety                                        

b. Depression  

c. Mania                                           

d. Schizophrenia

12. Lack of concentration is typically seen in   

a. Depression                                               

b. Mania         

c. Schizophrenia                              

d. All mental illness

13. Orientation to time, place and person is impaired in

a. Schizophrenia                              

b. Mania         

c. Depression                                    

d. Personality problems

14. Loss of memory among elders is a sign of

a. Alzheimer’s disease                    

b. Bipolar disease       

c. Parkinson’s disease                      

d. Adverse effects of medications

15. Repeating an act again and again is an indication of   

a. Normal Physical Health              

b. Normal Mental Health       

c. Mental Illness                               

d. Physical illness

16. Sleeplessness can cause

a. Diseases of eyes                          

b. Any physical illness

c. Social illness                               

d. Mental illness

17.  Inducing vomiting to lose weight is a sign of    

a. Hunger                                                           

b. mental illness

b. Diseases of the digestive tract                 

d. food baiting

18. People hide mental illness because of

a. Anger                                                        

b. Heavy costs

c. Social stigma                                            

d. Fear

19. The most important step to avoid mental illness is

a. Avoid mentally ill person                  

b. Earn lot of money   

c. Boycott the family of mentally ill      

d. Create awareness about mental illness

20. The most important step to treat mental illness is

a. Identify the mentally ill               

b. Show to doctor       

c. Give medications                          

d. Hospitalize the mentally ill

Appendix 2

**Table 5:** Checklist based on the Objective Structured Practical Examination (OSPE)

SR. N.	Skill Assessment Statements	Never	Once in a while	sometimes	Most of the times	Always
		0	1	2	3	4
1	Greets the patients appropriately					
2	Introduces self and establishes rapport					
3	Asks relevant questions to collect family history of mental illness.					
4	Enquires about present complaints					
5	Collects information regarding duration of symptoms					
6	Asks appropriate questions to gain information about the intensity of symptoms					
7	Scores correctly for the items that are not asked.					
8	Scores correctly for the items that are not responded by the patient.					
9	Is able to control own emotions while collecting information.					
10	Does not hesitate to ask questions related to sexual life, drug abuse and suicide information.					
11	Is confident in handling the GMHAT-PC Marathi version.					
12	Can use the GMHAT-PC Marathi version interview in sequential order.					
13	Collects relevant information from informants/family members before scoring.					
14	Interprets the information by patient correctly.					
15	Records information in the GMHAT-PC android version correctly.					
